# Obstetrics and Gynæcology

**Published:** 1899-06

**Authors:** Walter C. Swayne


### OBSTETRICS AND GYNÆCOLOGY.

**Post-partum hemorrhage** is truly described as *the* obstetric emergency in which prompt and proper action on the part of the attendant is of vital importance to the patient, as the issue will be determined before assistance can be obtained or complicated measures carried out. The rules for treatment therefore should be as simple and widely understood as possible. Too much stress cannot be laid on prophylactic measures. The third stage of labour should be left to nature, but not to nature unwatched and unassisted, the fundus being carefully controlled by light manual pressure until and for some time after the third stage is completed. Unnecessary hurry in this stage is to be deprecated; half an hour is quite an average time. Secondary uterine inertia from exhaustion by prolongation of the second stage and the withholding of nourishment should not be allowed to occur. If extractive manipulations are necessary, they should be conducted in a manner as much like the natural process as possible. In no case should the uterus be emptied between the pains, and the fundus should be followed down by the hand as the contents are expelled. Should inertia be dreaded, strychnia and ergotin, the latter in ¼-grain doses thrice daily, should be administered during the last month.

Delee[1] gives some novel methods of treatment in cases where *post-partum* hemorrhage occurs in spite of all precautions: in the first place, if the third stage has not been completed, and this will usually be the case when the hemorrhage is partly or entirely caused by laceration, the placenta must be at once

[1] *J. Am. M. Ass.*, 1899, xxxii. 802.

expressed or removed by the hand; the uterus being empty, firm bimanual compression is employed, doubling the uterus on itself and closing the cervix. He also recommends compression of the aorta. He then suggests, supposing that by compression the uterus is under control, that it should be plugged with iodoform, sterilised, or borated gauze; in order to do this the anterior and posterior lips are seized with vulsella and the uterus drawn down to the outlet: this proceeding itself is a valuable remedial agent, since traction on the cervix straightens out the vessels in the broad ligament and by putting the tissues on the stretch compresses them: three to four yards of the packing, which should be a yard wide, are then introduced into the uterine cavity until it is filled tightly; if after this proceeding any oozing takes place, he stitches the lips of the cervix together so that the uterine cavity is completely closed. He states that he has found this proceeding of great value.

He mentions the use of the hot douche at 120°F. after it has been ascertained whether the hemorrhage is due to atony or to laceration. We would prefer to reverse the order: the hot douche will almost invariably either stop or cause a great diminution of hemorrhage from either or both of these causes, especially that due to atony; the action of the douche is much increased by the use of half an ounce of ordinary vinegar to the pint; should this not cause cessation, lacerations are probably present, and the uterus should be packed while the lacerations are sewn up. The packing may be left in for twenty-four or forty-eight hours; but if iodoform gauze is used, watch must be kept for the symptoms of iodoform poisoning—namely, headache and rise of temperature, which, if present, will disappear at once on removing the packing.

The suture of the lesion seems rather a refinement: the slight oozing which occurs after packing is often due to uterine contractions squeezing out serum and blood that has been already absorbed by the packing, and if the latter is done sufficiently tightly the hemorrhage will infallibly cease. This method is undoubtedly of the greatest value, and should be employed more frequently as an emergency measure: the hot douche is preferable, as it can be used without removing the supporting and compressing bands for more than a moment, and does not require assistants in addition to the nurse usually present; in any case manual compression and the hot douche will give time for further skilled assistance to be obtained, when the packing can be done.

The sense of security in the mind of the attendant when he has packed the uterus tightly is most gratifying; there need then be no anxiety about recurrence of the bleeding, a state of things which under other circumstances will keep him in a state of anxiety for several hours. In the obstetric department of the Bristol Royal Infirmary this method is looked upon as a routine measure to be adopted in all cases where artificial emptying of the uterus is resorted to if hemorrhage occurs after it, especially in cases of abortion and miscarriage after the use of the curette. Not only is hemorrhage prevented, but involution is more rapid owing to the securing of the complete evacuation which occurs when the gauze is removed.

The **uterus** is one of the commonest organs to be attacked by **malignant disease**; in fact, one-third of the total cases of cancer occurring in the female consist of those in which this organ is attacked.

The nature of the treatment to be adopted depends on several factors in each case, the most important of which is the stage at which the disease has arrived when the question of the treatment to be adopted first arises, since on this depends very largely its success or failure. As regards treatment, we may at once exclude all other than radical operative measures except in those cases in which the disease is so far advanced that obviously no radical measures will give more than temporary relief, owing to the impossibility of removing the whole of the affected tissue. As regards operative measures, the broad lines of procedure may be briefly laid down as follows: Radical operation should not be undertaken unless there is a reasonable hope that all affected tissue can be removed without causing damage to vitally important organs, or without subjecting the patient to an operation of such severity as to render her immediate recovery unlikely.

Another most important point for consideration is the **prospect of cure**, or the length of time which will elapse after recovery from the operation before recurrence takes place. This practically depends on several conditions: firstly, the possibility of complete operative removal; secondly, the method of performance of the operation; and thirdly, the nature and situation of the growth. As regards operative treatment of these cases considered generally, the prospect here is apparently quite as good if not better than that in cases where other organs are affected which can be subjected to radical treatment, *e.g.*, the breast, tongue, lip, penis, or rectum. Recurrence, after operations on the uterus, does not take place any sooner than, if as soon as, after operations on the above, which are considered the most favourable situations for operative treatment; the immediate mortality is if anything lower than after operations on the above-named other organs; therefore operation may be undertaken under favourable conditions quite as readily.

Even in those cases where after radical operation undertaken for uterine cancer early recurrence takes place, unless this occurs in the peritoneum, the fatal termination is attended with less suffering, while in the interval the patient may be restored to comparative health by the removal of the sloughing mass, which causes a severe form of septic absorption, with its attendant wasting anæmia and pains.

Halliday Croom,[1] in a paper on **vaginal hysterectomy**, gives a very unfavourable account of his own results from this operation. He is so impressed with the rapidity of recurrence and the great pain suffered by those who die after this operation with secondary peritoneal infection, that he goes so far as to state that although in certain favourable cases recurrence may not occur, or only occur after some years, yet as a general rule recurrence occurs early, and if in the peritoneum the patient's exitus is much more painful than if the growth had not been attacked. It appears to me, however, that it is impossible to say how any given case will terminate; and while it is quite true that in certain cases the rate of progress of the growth is slow, and that the patient may live many months in a greater or less degree of discomfort, at the same time we cannot prophecy as to whether secondary infection of the peritoneum will not take place even if the growth is left alone, and while the fatal result is ultimately certain if the latter plan is adopted, it is equally certain that in cases subjected to radical treatment a certain number will live perhaps for several years in comparative health and comfort. Croom states that of the cases operated on by him, fourteen in number, all were dead within the year—a truly sombre picture, and gives a most gloomy view of the results of surgical intervention in these cases; he quotes the following results from the practice of others, thus: from the statistics of the Dresden Klinik forty-five cases out of eighty were free from recurrence two years after operation; thirty-four out of fifty-eight were without recurrence three years after; twenty-five out of forty-two, four years after; eighteen out of thirty, five years after; six out of nine, six years after; two cases who had survived seven years were quite well; while in Leopold's practice we are informed that seventy-two out of seventy-six of his cases were well and without recurrence from one to five years after operation.

It would appear that Croom takes too gloomy a view of the case, and that it is not justifiable to augur, as he does, that the operative treatment of cancer of the uterus is to be condemned because in a limited number of cases such as his the result as regards recurrence has not been good.

At the eleventh annual meeting of the Southern Surgical and Gynecological Association, held at Memphis, U.S.A., McMurtry[2] read a paper on the same subject, which has a striking similarity to that of Halliday Croom, and described five cases on which he had operated by vaginal hysterectomy during the past year, two of which had recurrence within five months, one in the bladder and one in the vagina; all five appeared to be typically suitable cases for the operation. He

[1] *Edinb. M. J.*, 1898, N.S. iv. 489. [2] *Am. J. Obst.*, 1899, xxxix. 217.

considered it doubtful whether one of the three remaining would be alive three years after operation. He advocated as a preferable measure the removal of the uterus by abdominal section, with a liberal removal of adjacent structures, especially the vaginal wall; he did not, however, advocate removal of the sub-peritoneal glands, as their enlargement was often due to inflammation.

In the subsequent discussion, which brought out strongly the opinion that the negro race is not so little susceptible to cancer as is generally supposed, Howard Kelly expressed surprise at the author's conclusions, and stated that his experience—a by no means small one—led him to quite an opposite opinion, many of his cases of vaginal hysterectomy for cancer having completely recovered and remained well for several years.

Against the gloomy views taken by McMurtry and Croom we may place the opinions and records of F. B. Jessett,[1] who publishes one hundred and seven cases distributed over seven years. We gather from his statements that nine died as the immediate result of operation. He prefaces a very interesting table with some instructive remarks on diagnosis and pathology; he lays great stress on the necessity for the vaginal examination of women who suffer from irregular hemorrhages apart from parturition and pregnancy, and especially of those who so suffer when past the climacteric, or who have any watery or fœtid discharge in addition to the hemorrhage: while this necessity is not recognised, a very large number of cases must go untreated until surgical intervention is useless except as a palliative. He regards malignant disease in its inception as being purely local, and considers that until cellular tissue is invaded the lymphatics are unaffected. As previously stated, nine operations had a fatal termination, not a large number considering the gravity of the operation, and this includes complicated cases. He considers that operation should be limited to those cases in which the mobility of the uterus is not impaired and the broad ligaments not invaded. He does not consider that invasion of the vaginal wall is an absolute contra-indication, nor that it is necessary that the cervix should be able to be drawn down to the outlet, a condition considered essential by some operators. He considers that affection of the iliac glands occurs late in the disease. As regards technique, he is of opinion that the union of the vesical and rectal peritoneal edges by suture is very important, and prefers to use the ligature for securing the uterine and ovarian vessels. He gives the following as the **indications for advising operation**: coloured and offensive discharge occurring in a patient at or past the menopause, and the passing of the sound causing bleeding; if after vaginal examination no growth is felt, dilatation of the cervix, exploration of the uterine cavity, curetting and careful microscopical examination of removed structures; if malignant growth is felt on

[1] *Brit. Gynæc. J.*, 1899, xiv. 541.

vaginal examination, or if the portions removed show malignant tissue under the microscope, he advises hysterectomy.

Of his 107 cases, nine died and recurrence took place in fifty-one, which number includes those lost sight of; forty-seven remained free from recurrence for periods varying from two years in the case of the last series to seven years in the case of the first; his figures as regards recurrence show a striking similarity to those of the Dresden Klinik mentioned above, the percentages being nearly the same. This seems to be, to a certain extent, an answer to Croom's statement, that cancer of the uterus in England and Germany must be two different things.

In the same way that the operation for removal of cancer of the breast has been extended so as to include the clearing of the axilla and removal of the subclavicular glands and tissue, with a marked improvement as regards delaying recurrence, operators are now seeking to apply the same principles to the uterus. To this end Freund's operation is being increasingly performed, with the additional procedure of removing a large part of the pelvic peritoneum together with the glands in the pelvis and the whole of the broad ligaments.

Ries [1] reports fifteen cases treated by the Ries-Clark operation, with three deaths. The **technique of the operation**, which is essentially a panhysterectomy by the combined method, is as follows:—The patient is prepared by a preliminary curetting and cauterisation under anæsthesia, while at the same time the whole pelvis is carefully explored for infected glands. If the growth is within the cervix, the cervical lips are sewn together to prevent contamination; if without, a vaginal cuff is raised and sewn over the cervix. Fresh assistants, instruments, sponges, etc., are provided for the next steps. The patient is placed in steep Trendelenburg position, incision from pubes to umbilicus, and the pelvis palpated carefully to exclude enlarged and immovable glands; these being found absent, the infundibulo-pelvic ligament is ligatured and cut close to the pelvic wall. The peritoneum is then incised along the common iliac artery, and the vessels exposed by dissection until the ureter is reached; the ureter is then exposed in the whole of its length in the pelvis —*i.e.* from the brim to the base of the bladder. Bleeding points are clamped as cut, or ligatured. When the uterine artery comes into view it is ligatured close to its origin, but outside the ureter and by sight; then the whole of the cellular tissue and glands lying between the external iliac artery superiorly, the pelvic wall laterally, the base of the bladder anteriorly, the pelvic floor inferiorly, and the meso-rectum posteriorly, is removed; the last lifted up and freed from all accessible glands. This procedure, begun on the left side, is repeated on the right. Adhesions between uterus and rectum are now divided if present. The round ligaments are secured, and the peritoneum of

[1] *Am. Gynæc. & Obst. J.*, 1898, xiii. 570.

Douglas's pouch incised close to rectum, and the vagina opened; the anterior space being already accessible as far as the peritoneum, the uterus is now free all around and is removed. The pelvis is now closed in by suturing the anterior edge of the peritoneum, which extends from the lateral wall across the vesico-uterine pouch, to the posterior which extends from the lateral wall across the pouch of Douglas; the abdomen is then closed in the ordinary way.

Werder[1] has devised a somewhat similar procedure, with this difference, that instead of by a prolonged dissection removing cellular tissue and glands, although he mentions this as an advantageous procedure, he removes the whole of the upper part of the vagina. He commences in the same way as Ries, but without freeing the peritoneum from the lateral walls of the pelvis, the anterior and posterior cul-de-sacs being opened and the lateral fornices separated from them. Recurrence is known to have taken place in forty-six cases; in twenty-four cases the average time before recurrence was nine months, and the average time before death sixteen months.

The result of an examination of the foregoing appears to be—(1) That the immediate mortality of all cases from the operation may be taken roughly as less than 10 per cent. if vaginal hysterectomy is chosen, but may be expected to be more if the more extensive operations of Ries, Clark, and Werder be undertaken; but if only the most favourable cases be selected, the mortality will not be higher than 7 to 8 per cent. (2) That a restoration to health of a certain duration may be expected in all cases; this may extend from six months to possibly permanent cure. (3) That recurrence will take place in less than two years in more than half the cases subjected to vaginal hysterectomy, and in the greater part of the remainder within five years. (4) That possibly 10 per cent. of cases will survive at the end of six years.

As regards the operations of Ries, Clark, and Werder, further experience is required. The principle seems surgically sound, but we must not expect very striking alterations in the recurrence-rate in view of the large number of cases in which this occurs in the scar, or peritoneum; its benefit is only likely to be gained by lessening the number of cases in which recurrence takes place in the glands, or by extension through and from cellular tissues already partly infected.

Finally, the possibility of this disease being present should never be lost sight of by the practitioner in any case where irregular hemorrhages, apart from the puerperal state, ovarian or fibroid tumours, are present, especially after the menopause, and all women should be kept informed of the importance of early diagnosis and the necessity for physical examination when suspicious symptoms are present. Out of the last fifty cases seen by me, only five were fit for radical operation; in two

[1] *Am. J. Obst.*, 1898, xxxvii. 289.

others an operation was possible, but not likely to give rise to other than the most temporary benefit. This seems to show an appalling state of ignorance, not on the part of the profession, since in all cases the disease had been already recognised, but on the part of the patients, on whom definite and distinct symptoms had not appeared to impress the necessity for seeking advice.

WALTER C. SWAYNE.

